# Impact of Methylglyoxal and High Glucose Co-treatment on Human Mononuclear Cells

**DOI:** 10.3390/ijms10041445

**Published:** 2009-03-31

**Authors:** Ming-Shu Hsieh, Wen-Hsiung Chan

**Affiliations:** Department of Bioscience Technology and Center for Nanotechnology, Chung Yuan Christian University, Chung Li, Taiwan 32023

**Keywords:** High glucose, methylglyoxal, apoptosis, ROS, nitric oxide

## Abstract

Hyperglycemia and elevation of methylglyoxal (MG) are symptoms of diabetes mellitus (DM). In this report, we show that co-treatment of human mononuclear cells (HMNCs) with MG (5 μM) and high glucose (HG; 15 – 30 mM) induces apoptosis or necrosis. HG/MG co-treatment directly enhanced the reactive oxygen species (ROS) content in HMNCs, leading to decreased intracellular ATP levels, which control cell death via apoptosis or necrosis. Concentrations of 5 μM MG and 15 mM glucose significantly increased cytoplasmic free calcium and nitric oxide (NO) levels, loss of mitochondrial membrane potential (MMP), activation of caspases-9 and -3, and cell death. In contrast, no apoptotic biochemical changes were detected in HMNCs treated with 5 μM MG and 25 mM glucose, which appeared to undergo necrosis. Pretreatment with nitric oxide (NO) scavengers inhibited apoptotic biochemical changes induced by 5 μM MG/15 mM glucose, and increased the gene expression levels of p53 and p21 involved in apoptotic signaling. The results collectively suggest that the treatment dosage of MG and glucose determines the mode of cell death (apoptosis vs. necrosis) of HMNCs, and that both ROS and NO play important roles in MG/HG-induced apoptosis.

## 1. Introduction

Hyperglycemia and increased methylglyoxal (MG) levels, two major symptoms of DM, have longterm effects on blood and endothelial cells of blood vessels [1, 2]. Previous reports show that high glucose (HG) and MG cause various cell injuries, including apoptosis [2–4]. A recent study further demonstrates that co-treatment of cells with MG and HG directly evokes ROS generation. The amount of ROS generated depends on the treatment dosage, and determines the intracellular ATP level, which regulates the cell death mode [5]. Additionally, calcium influx and NO are involved in HG/MGinduced apoptosis of human umbilical vein endothelial cells (HUVECs) [5]. Both endothelial and blood cells may encounter these injury factors in the blood circulation of DM patients. However, the combined effects of MG and hyperglycemia in blood cells, and the underlying signaling pathways, remain to be elucidated.

High glucose promotes a hyperosmotic shock environment, leading to osmotic stress that triggers various cell responses, including apoptosis [3, 6, 7]. Hyperosmotic shock induces apoptosis through multiple biochemical changes, including ROS generation, activation of JNK and caspase-3, and DNA fragmentation. These effects are blocked by antioxidants, suggesting that ROS play important roles in hyperglycemia-induced apoptosis [3]. Further studies show that HG-induced apoptosis is mediated by oxidative stress and JNK activation [2, 3]. However, the effects of the hyperglycemic environment on blood cells are yet to be evaluated.

MG, a reactive dicarbonyl compound formed as a metabolic byproduct of glycolysis, is produced in various foodstuffs during processing, including freshly brewed coffee and soy sauce [8]. Coffee is highly consumed worldwide, and intake of MG via ingestion of 2–3 cups of coffee can be as much as 1 mg/day [8]. Moreover, MG levels in diabetics remain elevated for several months, even years. High levels of MG are often observed in the blood of diabetic patients, leading to serious toxicological effects. Methylglyoxal (MG) is a known metabolic product of glucose. Under hyperglycemic conditions, MG is formed from triose phosphate or acetol, and serves as a precursor to advanced glycation-end products (AGEs) [9–11]. In diabetic patients, the serum concentration of MG or MG-derived AGEs is significantly increased compared to that in non-diabetics, and MG has been implicated in diabetes-associated diseases including hypertension, retinopathy and nephropathy [12–14]. Since diabetic complications develop at a slow rate, the long-term effect of MG on AGE formation has been investigated in detail [15, 16]. In addition, MG modifies cross-linked lysine and arginine residues, altering the protein characteristics and producing reactive oxygen species (ROS) during the glycation reaction[17–19]. Oxidative stress and AGE formation are associated with impaired cognitive processes in diabetic patients [20, 21], suggesting linkage to MG toxicity. Recent studies show that MG induces apoptosis in several cell lines, including Schwann, PC-12, and renal tubular cells [22–24]. However, the cytotoxic effects and regulatory mechanisms of MG on blood cells require further investigation.

Numerous chemical and physical treatments capable of inducing apoptosis stimulate oxidative stress via generation of ROS in cells [25–27], suggesting a close relationship between oxidative stress and apoptosis. Nitric oxide (NO) is an important second messenger involved in a variety cellular responses and biological functions, including tumor development, metastasis and apoptosis [28–30]. Recent studies demonstrate that NO is largely produced in mitochondria through the actions of a Ca^2+^- sensitive mitochondrial NO synthase (NOS) [31, 32]. NOS-mediated NO production may control oxygen consumption and mitochondrial membrane potential through cytochrome c oxidase. The NO molecule is subsequently reactivated with superoxide to produce peroxynitrite, further modifying its target substrates and inducing oxidative stress [33–35]. Oxidative stress and Ca^2+^ influx act as upstream regulators of mitochondrial NOS activity [36, 37]. Moreover, our group recently showed that MG/HG co-treatment triggers NOS activation, and that NO generation and calcium influx are essential for MG/HG-induced apoptosis of HUVECs [5]. However, the biochemical events involved in MG/HG-induced cell injury and the regulatory mechanisms in blood cells underlying these effects are currently unclear.

Apoptosis and necrosis, two distinct cell death types, have different biochemical and morphological characteristics and regulatory mechanisms [38–42]. Apoptosis plays an important role in the embryogenesis and homeostasis of multicellular organisms, and impairment of apoptotic function is associated with several human diseases, including neurodegenerative disorders and cancer [43]. In contrast, necrosis occurs in response to acute and nonphysiological injury [44]. Recent reports indicate that the magnitude of initial insult, and not stimulus type, plays a critical role in prompting cells to undergo either necrosis or apoptosis [41, 45, 46]. The ‘choice’ between necrosis and apoptosis is possibly controlled by intracellular ATP levels and/or caspase inactivation [47]. In particular, high energy levels are required for execution of apoptotic processes, whereas necrosis can proceed in the presence of low ATP levels [41, 42, 48, 49]. Here, we focus on the effects of ROS and intracellular calcium influx on NO production, and the modulatory effects on cell death modes triggered by treatment of HMNCs with a combination of MG/HG.

### 2. Results and Discussion

**Figure 1.**
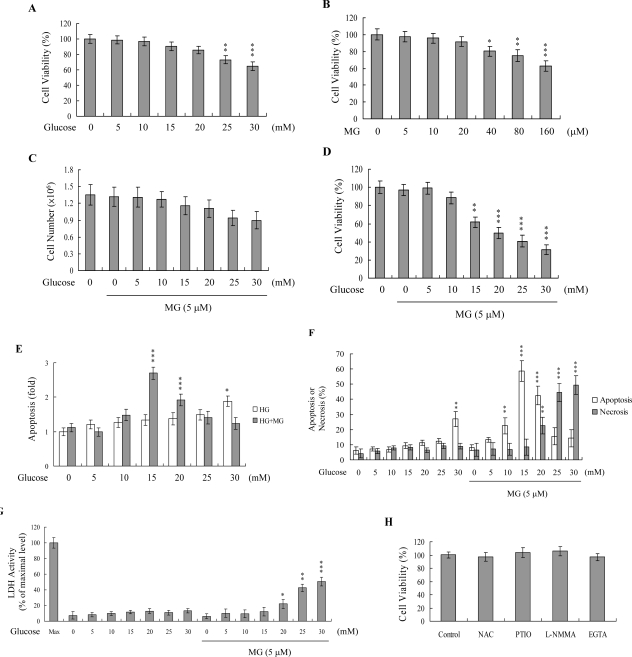
Effects of MG and HG on HMNCs. (A and B) HMNCs were incubated with 0 – 30 mM glucose (A) or 0 – 160 μM MG (B) for 24 h. Cell viability was determined using the MTT assay. (C-G) HMNCs were treated with or without MG (5 μM) and various concentrations of glucose for 24 h. Total cell numbers after treatment were measured by trypan blue staining (C). Cell viability was measured (D). Apoptosis was detected with the Cell Death Detection ELISA kit (E), followed by staining with propidium iodide and Hoechst 33342 (F). Necrosis was further assessed in terms of LDH activity released in the culture medium, with data expressed as a percentage of the maximal level (Max) of LDH activity determined after total cell lysis (G). (H). HMNCs were incubated with N-acetyl cysteine (NAC; 300 μM), l-NMMA (400 μM), PTIO (20 μM) and EGTA (500 μM) for 24 h. Cell viability was determined using the MTT assay. Values are presented as means ± SD of eight determinations. *P < 0.05, **P < 0.01 and ***P < 0.001 versus the untreated control group.

To determine the combined effects of MG and hyperglycemia, HMNCs were simultaneously treated with MG and HG, and cell viability examined. Importantly, co-treatment with MG and glucose at concentrations similar to those detected in the plasma of DM patients (5 μM and 15 mM, respectively) decreased cell viability, but not treatment with either agent alone (Figures 1A-D). To determine the cell death mode underlying this decreased viability, cellular apoptosis and necrosis were evaluated using the TUNEL assay, Propidium Iodide/Hoechst 33342 staining, and LDH activity assay. Interestingly, HMNCs treated with 5 μM MG and 15–20 mM glucose showed a marked increase in the apoptotic percentage, whereas those treated with 5 μM MG and concentrations of glucose higher than 20 mM displayed a significantly increased necrotic cell population (Figures 1E–1G). Our results suggest that a co-treatment of HMNCs with MG and HG at concentrations similar to those observed in the blood circulation of DM patients promotes injury via apoptotic and necrotic processes. In view of our previous findings that numerous stimuli, including MG and HG, trigger apoptosis via ROS generation [3, 4], we used DCF-DA and DHR 123 to examine ROS formation in HMNCs co-treated with MG and HG.

**Figure 2.**
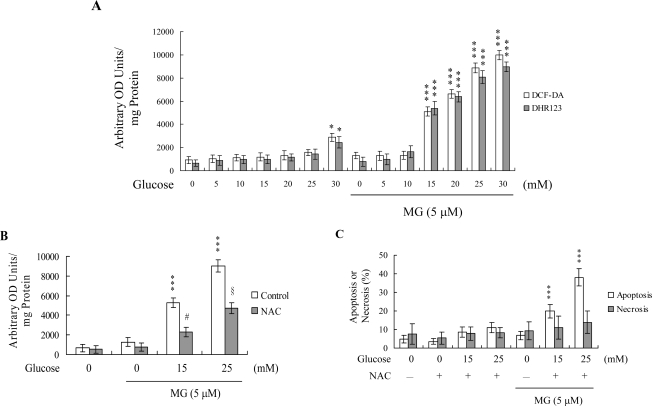
MG/HG co-treatment promotes oxidative stress in HMNCs. (A) HMNCs were treated with or without MG (5 μM) and various concentrations of glucose for 24 h. ROS generation was assayed using DCF-DA (20 μM) or dihydrorhodamine 123 (DHR 123; 20 μM) dye. (B and C) HMNCs were pre-incubated with N-acetyl cysteine (NAC; 300 μM) for 30 min, followed by treatment with or without MG and glucose, as indicated. ROS generation was assayed using DCF-DA dye (B). The percentage of apoptosis or necrosis (C) was measured, as described in Figure 1. Data are representative of eight independent experiments. ***P < 0.001 versus the untreated control group. #P < 0.001 versus the “MG + HG (15 mM)” group. §P < 0.001 versus the “MG + HG (25 mM)” group.

A combination of 5 μM MG and 15 – 30 μM HG stimulated ROS generation about 5.5 – 11-fold, compared to the untreated control group (Figure 2A). Interestingly, pretreatment with *N*-acetylcysteine (NAC), a commonly used ROS scavenger, effectively prevented ROS generation by 5 μM MG and 15 mM glucose, and partially blocked ROS production by 5 μM MG and 25 mM glucose (Figure 2B). Furthermore, NAC pretreatment inhibited cell apoptosis induced by 5 μM MG/15 mM glucose, and shifted the mode of cell death stimulated by 5 μM MG/25 mM glucose from necrosis to apoptosis (Figure 2C). These results suggest that the ROS content participates in determining the cell death mode of MG/HG-treated HMNCs.

**Figure 3.**
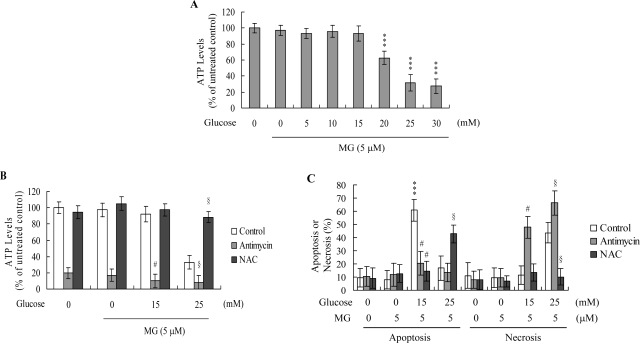
MG/HG co-treatment induces a decrease in intracellular ATP levels. (A) HMNCs were treated with MG (5 μM) and various concentrations of glucose for 24 h. Intracellular ATP levels were determined using a bioluminescence-based ELISA assay. Data are presented as a percentage of untreated controls, and values as means ± SD of five determinations. (B and C) HMNCs were incubated with antimycin (2 μM) or NAC (300 μM) for 1 h, followed by treatment with MG and glucose, as indicated, for a further 24 h. ATP levels (B) and percentage of cell apoptosis or necrosis (C) were measured. The given values are representative of eight determinations. ***P < 0.001 versus the untreated control group. #P < 0.001 versus the “MG + HG (15 mM)” group. §P < 0.001 versus the “MG + HG (25 mM)” group.

We previously demonstrated that the ATP level is an important mediator capable of switching the mode of cell death from apoptosis to necrosis [41]. To further elucidate the mechanism by which MG/HG triggers either apoptotic or necrotic processes, we examined the ATP content in MG/HGtreated HMNCs. Treatment of HMNCs with 5 μM MG and 20 – 30 mM glucose induced a decrease in the ATP level, whereas 5 μM MG and 5 – 15 mM glucose had no effect on the cellular ATP content (Figure 3A). To further clarify the role of intracellular ATP levels in MG/HG-induced apoptosis or necrosis, we examined the cell death modes of ATP-depleted cells. HMNCs were treated with antimycin (a mitochondrial inhibitor), which causes ATP depletion (Figure 3B) or left untreated, and the modes of MG/HG-induced cell death examined. Pretreatment of cells with antimycin led to switch of apoptosis induced by 5 μM MG and 15 mM HG to necrosis, and enhanced 5 μM MG and 25 mM glucose-stimulated necrosis (Figure 3C). Moreover, NAC pretreatment effectively blocked the 5 μM MG and 25 mM glucose-induced decrease in ATP levels, and induced a subsequent switch from necrosis to apoptosis (Figures 3B and 3C), suggesting that the ROS content mediates intracellular ATP levels and modulates the associated switch between apoptosis and necrosis in MG/HG-treated HMNCs.

**Figure 4.**
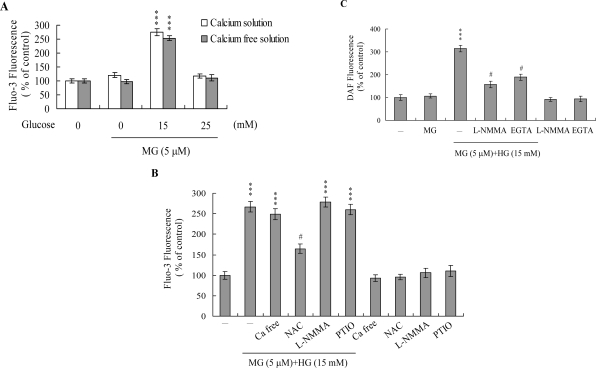
MG/HG co-treatment triggers changes in intracellular calcium and NO content in HMNCs. (A) HMNCs were incubated with MG and glucose, as indicated, for 24 h. Intracellular Fluo-3 fluorescence intensity was measured in the presence/absence of extracellular Ca^2+^. (B) Intracellular Ca^2+^ level changes triggered by co-treatment with MG (5 μM) and HG (15 mM), and effects of ROS and NO inhibitors (NAC: 300 μM; l-NMMA: 400 μM; PTIO: 20 μM). (C) HMNCs were pretreated with L-NMMA (400 μM) or EGTA (500 μM) for 30 min, followed by incubation with or without MG (5 μM) and HG (15 mM). Intracellular NO generation was measured using the DAF-2DA fluorescence dye. Data are presented as a percentage of the control group. ***P < 0.001 versus the untreated control group. #*P* < 0.001 versus the “MG + HG” group.

Changes in [Ca^2+^]i in MG/HG-treated HMNCs were detected using the Fluo-3AM fluorescence dye. Treatment with 5 μM MG and 15 mM glucose elicited an increase in [Ca^2+^]i, but not 5 μM MG and 25 mM glucose (Figure 4A). Furthermore, cells cultured in Ca^2+^-containing medium displayed a ~2.7-fold increase in [Ca^2+^]i following treatment with 5 μM MG and 15 mM glucose, while no effects were evident on cells cultured in Ca^2+^-free culture medium (Figure 4A). These findings indicate that the rise in [Ca^2+^]i is primarily attributed to the influx of internal Ca^2+^, such as that observed in the endoplasmic reticulum, mitochondria, nucleus and/or calcium-binding proteins (Figure 4A).

PTIO, an inhibitor of NOS and scavenger of NO, and L-NMMA, an inhibitor of NO synthase (NOS), had little effect on the [Ca^2+^]i increase induced by 5 μM MG and 15 mM glucose, whereas pretreatment with NAC significantly suppressed this increase (Figure 4B). These results suggest that elevation of [Ca^2+^]i induced by 5 μM MG/15 mM glucose is regulated by ROS, but not NO. We further employed the NO-sensitive dye, DAF-2DA, to measure intracellular NO generation during MG/HG-induced apoptosis. Intracellular NO levels were increased in HMNCs co-treated with 5 μM MG and 15 mM glucose (Figure 4C). However, this increase was prevented upon pretreatment of cells with the NOS inhibitor, l-NMMA (Figure 4C) or 500 μM EGTA (a Ca^2+^ chelator) (Figure 4C). Our results suggest that intracellular Ca^2+^ levels play an important role in NOS activation and NO increases observed in HMNCs co-treated with 5 μM MG and 15 mM glucose.

**Figure 5.**
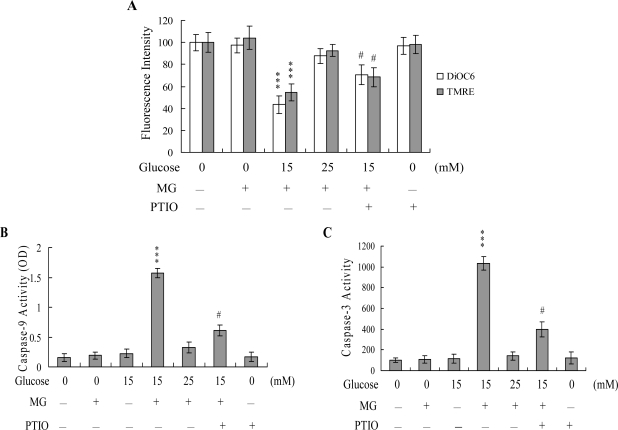
MMP changes and activation of caspase-9 and -3 following MG/HG treatment of HMNCs. HMNCs were pretreated with PTIO (20 μM) for 1 h, and treated with MG (5 μM) and the indicated concentrations of glucose for another 24 h. (A) Mitochondrial membrane potential changes was analyzed using 40 nM DiOC6(3) or 1 μM TMRE. (B) Caspase-9 activity was assayed using the Colorimetric Caspase-9 Assay kit. (C) Caspase-3 activity was analyzed using Z-DEVD-AFC as the substrate. ***P < 0.001 versus the untreated control group. #*P* < 0.001 versus the “MG + HG (15 mM)” group.

Next, we analyzed changes in MMP, a major apoptotic event during mitochondrial-mediated apoptosis. Uptake of DiOC6(3) and TMRE into mitochondria of HMNCs was observed, indicating a significant loss of MMP following co-treatment with 5 μM MG and 15 mM glucose, which trigger apoptotic processes, but not 5 μM MG and 25 mM glucose, which induce necrotic processes (Figure 5A). In addition, we monitored activation of caspases-9 and -3 involved in mitochondrial-mediated apoptotic pathways. Treatment of HMNCs with 5 μM MG and 15 mM glucose stimulated the activation of caspases-9 (Figure 5B) and -3 (Figure 5C), but not 5 μM MG and 25 mM glucose. Importantly, both loss of MMP and caspases activation were significantly inhibited upon incubation of cells with 20 μM PTIO, prior to co-treatment with 5 μM MG and 15 mM glucose (Figures 5A–5C). These results indicate that the increase in the NO level may act as an upstream regulator of MMP changes and activation of caspase-9 and -3 during MG/HG-induced apoptosis, but not necrosis of HMNCs.

**Figure 6.**
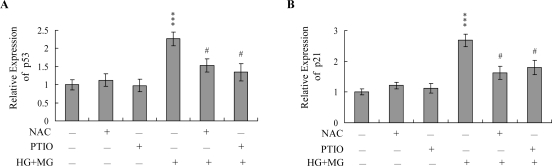
Effects of NAC and PTIO on the mRNA levels of p53 and p21. HMNCs were pre-incubated with NAC (300 μM) and PTIO (20 μM) for 1 h or left untreated, followed by treatment with MG (5 μM) and HG (15 mM) for another 24 h. The mRNA levels of p53 (A) and p21 (B) were analyzed using real-time PCR. Values are representative of eight determinations. ***P < 0.001 versus the untreated control group. #*P* < 0.001 versus the “MG+HG” group.

Data from real-time RT-PCR analyses revealed significant upregulation of p53 and p21 mRNA in HMNCs co-treated with 5 μM MG and 15 mM glucose, which was blocked by pretreatment with NAC or PTIO (Figures 6A and B).

**Figure 7.**
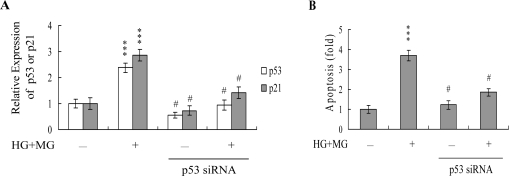
Knockdown of p53 protects HMNCs against MG/HG-induced apoptosis. HMNCs were transfected with siRNA targeting p53, incubated for 24 h, and treated with MG (5 μM) and HG (15 mM) for a further 24 h. (A) The mRNA levels of p53 and p21 were analyzed using real-time PCR. (B) Cell apoptosis was measured as described in Figure 1. ***P < 0.001 versus the untreated control group. #*P* < 0.001 versus the “MG + HG” group.

To further determine the roles of p53 and p21 in MG/HG-induced apoptosis, we used targeted siRNAs to suppress p53 expression in HMNCs. Cells were incubated with 5 μM MG and 15 mM glucose for 24 h, and tested for viability. Knockdown with p53 siRNA led to a significant decrease in the mRNA levels of p53 and p21 in MG/HG-treated HMNCs (Figure 7A), which was associated with a marked decrease in MG/HG-induced apoptosis (Figure 7B). These results suggest that MG/HG combination treatment upregulates p53 and p21 in HMNCs, subsequently promoting apoptosis of treated cells.

Increased concentrations of MG and glucose in blood are two major symptoms of DM. We previously showed that treatment with glucose at concentrations higher than 25 mM triggers apoptotic processes in K562 cells, a human leukemia cell line [3], and 250 μM MG causes various cells injuries, including apoptosis [4]. In addition, while the injury effects of MG/HG on HUVECs and the precise signaling cascades of cell death have been investigated [5], the influence of MG/HG on blood cells, particularly white blood cells involved in the immune defense system of DM patients, are unclear at present. Our present results indicate that glucose alone at concentrations higher than 25 mM and MG alone at concentrations higher than 40 μM induce a decrease in cell viability of HMNCs (Figures 1A and 1B). However, since hyperglycemia and increased MG levels are observed simultaneously in DM patients, we investigated the effects of co-treatment with MG and glucose. Co-treatment of HMNCs with 5 μM MG and 15 – 30 mM HG stimulated a significant decrease in cell viability (Figure 1D). Furthermore, within this range, the glucose concentration determined whether cell death occurred via apoptosis or necrosis (Figures 1E–G).

Earlier studies demonstrate that HG promotes ROS generation in human aortic endothelial [50] and K562 cells [3], and triggers apoptosis in HMNCs [51]. Additionally, a recent investigation shows that intracellular ROS is generated upon MG treatment of mouse embryonic stem cells [4]. MG treatment can increase intracellular ROS levels in several ways: (1) the glycation reaction of amino acids or proteins with MG can produce ROS (such as O_2_^−^); (2) metabolism of MG may deplete the cells’ glutathione content, thereby decreasing the elimination of intracellular ROS; and/or (3) ROS scavenger enzymes, such as superoxide dismutases, glutathione peroxidases and glutathione transferases, may be modified/inactivated by MG [17, 52, 53]. In addition, our recent studies have demonstrated that high MG levels may be the main cause of immune dysfunction in diabetic patients [18, 19]. We monitored DNA strand breakage, ROS generation and oxidative DNA damage induced *in vitro* by a 50 μM MG/lysine glycation reaction (3 h) in HMNCs. Our results revealed that oxidative stress and DNA damage were directly induced by 50 μM MG/lysine, indicating that oxidative stress plays a role in MG-induced cell injury [18]. Importantly, our group previously reported that methylglyoxal and high glucose synergistically trigger ROS generation to stimulate downstream apoptotic biochemical changes [5]. The present results disclose that co-treatment with MG/HG stimulates ROS generation to a higher extent than MG or HG alone (data not shown), implying that MG/HG act synergistically to produce ROS. The ROS generation mechanisms of methylglyoxal and high glucose are distinct. Mechanisms underlying the effects of a combination of MG/HG on ROS generation are more complex than those of MG and HG alone, and are under investigation in our laboratory at present. However, cells treated with 5 μM MG and co-treated with concentrations of glucose higher than 15 mM displayed significantly increased ROS generation (Figure 2A). The ROS content in these cells aided in determining whether subsequent cell death proceeded through apoptosis or necrosis (Figure 2C). These results signify that MG and glucose synergistically cooperate to promote ROS generation and cell apoptosis or necrosis.

Chemically induced hypoxia activates distinct types of cell death, depending on the intensity of the stimulus or triggering insult in rat fibroblastic cells [54]. Apoptosis was predominant in cells treated with low doses of antimycin A, a specific inhibitor of the mitochondrial respiratory chain, while necrosis was increasingly favored with increasing intensity of hypoxic insult [54]. Other studies suggest that the intracellular ATP level represents an important regulator of the switch to apoptosis or necrosis [41, 47, 49, 54], with necrosis favored under low ATP conditions in many model systems. Consistent with this theory, several studies show that high ATP levels are essential for nuclear morphologic changes characteristic of apoptosis [55]. Previously, we showed that 50 – 200 μM and 12.5 – 25 μM curcumin induced two distinct cell death programs in osteoblasts. In this system, curcumin decreased the intracellular ATP levels in osteoblasts in a dose-dependent manner, leading to an effective switch from apoptosis to necrosis [41]. According to these results, higher ROS generation induced lower intracellular ATP levels and necrotic death, while lower ROS levels during apoptotic stimulus treatment allowed cells to maintain a higher ATP level that induced cell death via apoptotic processes. In the present study, we show that the ROS content of MG/HG-treated HMNCs affects the intracellular ATP levels and aids in determining the mode of induced cell death (Figure 3). Previous findings, in conjunction with the present results, support the theory that the ROS content and ATP level act as the switch for determining whether MG/HG-induced cell death in HMNCs proceeds through apoptosis or necrosis.

Intracellular calcium levels play an important role in regulation of cell death [31, 56, 57]. In this investigation, we determine whether the MG/HG combination induces apoptosis through intracellular calcium increase. We observed an increase in [Ca^2+^]i following MG/HG treatment, which was largely attributed to influx of internal Ca^2+^ storage organelles (Figures 4A and 4B). This effect was significantly blocked by NAC (Figure 4B), indicating that MG/HG-induced ROS generation is responsible for elevation of intracellular calcium concentrations in treated HMNCs.

NO is an endogenous product of NADPH, O_2_ and l-arginine catalyzed by nitric oxide synthase. A recent study demonstrates that NO is involved in apoptosis triggered by several types of stimuli [31, 32]. The regulatory actions of NO on the mitochondrial apoptotic signaling pathways are well documented. Decreased ratios of Bcl-2/Bax and inhibition of electron transport are regulatory mechanisms of NO-mediated apoptosis [58]. A decrease in the Bcl-2/Bax ratio and impairment of mitochondrial electron transport may cause release of cytochrome c involved in the control of cell apoptosis. Additionally, tamoxifen enhanced the intramitochondrial Ca^2+^ concentration, leading to stimulation of mitochondrial NO synthase activity and NO production in rat livers and human breast cancer MCF-7 cells [32]. Here, we observed NO generation following MG/HG treatment of HMNCs, with ~ 3.1-fold higher intracellular NO levels in treated cells versus untreated controls (Figure 4C). Pretreatment with EGTA prevented this increase in intracellular NO to a marked extent (Figure 4C), indicating that NO production in MG/HG-treated HMNCs is dependent on the intracellular calcium concentration. The regulatory role of NO in cell apoptosis is complex, and several studies show that NO-mediated apoptotic effects are modulated by different mechanisms in distinct cell types [30, 59]. NOS substrates or NO donors inhibit photodynamic treatment-induced apoptosis in CCRF-CEM cells [60]. In our experiments, PTIO attenuated loss of MMP and decreased caspases activation (Figure 5), suggesting that NO is an important mediator of apoptosis in MG/HG-treated HMNCs.

NO-mediated apoptotic processes are associated with p53 gene activation, which is essential for regulation of the cell cycle and/or apoptotic signaling occurring through p21^Waf1/Cip1^ or Bax [61, 62]. Here, we show upregulation of both p53 and p21 mRNA expression following treatment with MG/HG, which was blocked upon pretreatment with PTIO and NAC (Figure 6). The p53 protein, which is the expression product of the *p53* tumor suppressor gene, prevents cell with damaged DNA from proliferating. The damaged DNA triggers the activation of an enzyme called ATM kinase, which catalyzes the phosphorylation of p53 proteins. Accumulation of phosphorylated p53 proteins in a DNA-damaged cell can activate two types of events: cell cycle arrest and cell apoptosis. The p53 protein is a transcription factor that can activate specific genes, including the *p21* gene. The p21 protein, a member of the Cdk inhibitor, can block the activation of the Cdk-cycline complex, thereby causing cell cycle arrest. The p53 protein can also activate genes encoding proteins that can trigger cell apoptotic processes through binding and inactivation of the Bcl2 protein, an inhibitor of apoptosis. In the present study, we found that siRNA-mediated knockdown of p53 mRNA prevented the MG/HGinduced up-regulation of *p21* mRNA expression, and decreased subsequent apoptosis (Figures 7A and B). These results indicate that p53 and p21 are activated during MG/HG-induced apoptosis of HMNCs. We hypothesize that, in MG/HG-treated HMNCs, generated ROS may trigger DNA damage, thereby activating p53 and triggering downstream apoptotic signal cascades. However, future studies will be required to examine the precise regulatory roles of p53 and p21 in MG/HG-induced apoptosis of HMNCs.

## 3. Conclusions

Co-treatment with MG/HG causes cell death (apoptosis or necrosis) in HUVECs [5]. However, no injury effects are observed upon treatment with MG or HG alone. Here, we further confirm this synergistic injury effect of MG/HG in blood cells.

**Figure 8.**
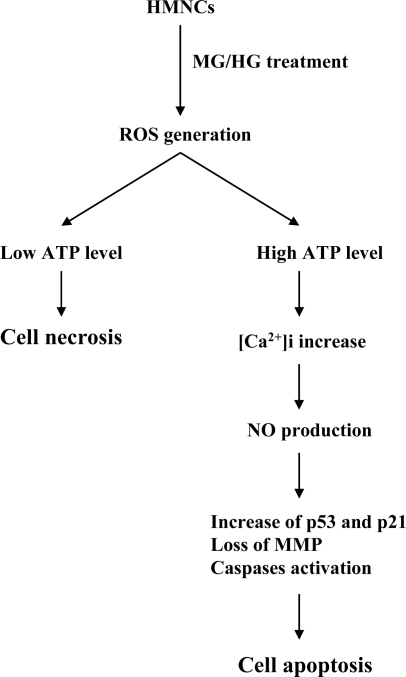
Scheme of events occurring during MG/HG-induced cell death in HMNCs.

It is possible that injury of DM patients affects endothelial cells of blood vessels and blood cells of circulation. However, the injury effects of MG/HG on DM patients *in vivo* require further investigation. Based on the results of the present study, we propose a possible model of MG/HGinduced cell injury signaling in HMNCs (Figure 8). Co-treatment of cells with MG and HG directly induces ROS, with the treatment dosage affecting the amount of ROS generated. The ROS content determines intracellular ATP levels, which regulate cell death, with high ATP levels favoring apoptosis and low ATP levels favoring necrosis. In addition, high levels of intracellular ROS trigger an influx of calcium from the intracellular Ca^2+^ storage organelles, leading to increased intracellular calcium concentrations that stimulate NO generation. Pretreatment with NAC or PTIO blocks the MG/HG-induced up-regulation of critical genes, and rescues cell viability. Our results collectively demonstrate that both intracellular ROS and NO play critical roles in MG/HG-induced apoptosis of HMNCs.

## 4. Experimental Section

### 4.1. Materials

3-(4,5-Dimethylthiazol-2-yl)-2,5-diphenyltetrazolium bromide (MTT), 2-phenyl-4,4,5,5-tetramethylimidazoline-1-oxyl-3-oxide (PTIO), *N*-acetylcysteine (NAC), 2’,7’-dichlorofluorescein diacetate (DCF-DA), propidium iodide, Hoechst 33342 and ethyleneglycol-bis(β-aminoethylether)tetraacetic acid (EGTA) were purchased from Sigma (St. Louis, MO). Z-DEVD-AFC was obtained from Calbiochem (La Jolla, CA).

### 4.2. Isolation of human mononuclear cells (HMNCs) and treatment

Blood was collected from 30 healthy humans (15 males and 15 females, all 20 years of age) who reported no history of smoking, allergies, and medication and alcohol consumption. Mononuclear cells were isolated from 50 mL aliquots of human blood using Histopaque 1077 [63]. The samples were centrifuged at 400 × g for 30 min, and HMNCs were collected from the interface and resuspended in Tris-buffered saline (20 mM Tris, 0.15 M NaCl, pH 7.4). The cells were sedimented by centrifugation at 300 × g for 15 min and then resuspended in RPMI. Isolated HMNCs from different humans were pooled and randomly selected for experiments. For experiments, cells were incubated with medium containing the indicated concentrations of MG and glucose. High glucose treatment was performed by incubating cells in medium containing various concentrations of glucose (5 – 30 mM) for 24 h. The osmolarity of medium (an isotonic solution) was 300 mosmol/kg. Following the addition of 5 – 30 mM glucose into the medium, the osmolarity of this hypertonic solution was altered to 305 – 330 mosmol/kg. The MG/HG co-treated cells were washed twice with ice-cold phosphate-buffered saline (PBS) and lysed on ice for 10 min in 400 μL lysis buffer (20 mM Tris-HCl, pH 7.4, 1 mM EDTA, 1 mM EGTA, 1% Triton^®^ X-100, 1 mM benzamidine, 1 mM phenylmethyl-sulfonyl fluoride, 50 mM NaF, 20 μM sodium pyrophosphate and 1 mM sodium orthovanadate). The cell lysates were sonicated on ice for 3 × 10 sec and centrifuged at 15,000 × g for 20 min at 4°C, and the supernatants were used as cell extracts.

### 4.3. MTT assay

Cell survival was monitored using the MTT (3-[4,5-dimethylthiazol-2-yl] -2,5-diphenyltetrazolium bromide) test. Briefly, cells were treated with the indicated concentrations of MG and glucose for 24 h, and then treated with 100 μL of 0.45 g/L MTT solution. The cells were incubated at 37°C for 60 min to allow color development, and then 100 μL of 20% SDS in DMF:H_2_O (1:1) solution was added to each well to stop the reaction. The plates were incubated overnight at 37°C for solubilization of the formazan products, and spectrophotometric data were measured using an ELISA reader at a wavelength of 570 nm.

### 4.4. Assessment of necrosis and apoptosis

Oligonucleosomal DNA fragmentation (a hallmark of apoptosis) was measured using the Cell Death Detection ELISA^plus^ kit (Roche Molecular Biochemicals, Mannheim, Germany). Cells (1 × 10^5^) were treated with or without the indicated concentrations of MG and glucose at 37 °C for 24 h, the procedures were performed according to the manufacturer’s protocol, and spectrophotometric data were obtained using an ELISA reader at 405 nm. In addition, cells were incubated with propidium iodide (1 μg/mL) and Hoechst 33342 (2 μg/mL) at room temperature for 10 min, and fluorescent microscopy was used to identify the percentage of propidium iodide-impermeable cells having condensed/fragmented nuclei (apoptotic) and the percentage of propidium iodide-permeable cells (necrotic). In each experiment, 8 – 10 independent fields (~ 500 – 800 nuclei in total) were counted per condition. The activity of lactate dehydrogenase (LDH) present in the culture medium was evaluated as an additional index of necrosis, as previously described [18, 42, 64, 65]. Briefly, cells (5 × 10^4^) were cultured in 96-well microtiter plates (100 μL medium/well), LDH activity was assayed using, and the absorption values at 490 nm were determined with an ELISA reader, according to the manufacturer’s instructions (Promega, Madison, WI). Blanks consisted of test substances added to cell-free medium.

### 4.5. ROS assay

ROS were measured in arbitrary units using 2’,7’-dichlorofluorescein diacetate (DCF-DA) or dihydrorhodamine 123 (DHR 123) dye. Cells (1.0 × 106) were incubated in 50 μL PBS containing 20 μM DCF-DA or DHR123 for 1 h at 37°C, and relative ROS units were determined using a fluorescence ELISA reader (excitation 485 nm, emission 530 nm). An aliquot of the cell suspension was lysed, the protein concentration was determined, and the results were expressed as arbitrary absorbance units/mg protein.

### 4.6. ATP level analysis

Cells treated with the indicated concentrations of MG and glucose were collected, and ATP levels were quantified with an ATP determination kit. Cells were collected by centrifugation, resuspended in distilled water and then boiled for 5 min. The ATP level in each extract was determined by a bioluminescence assay using a liquid scintillation analyzer, according to the manufacturer’s protocol (Molecular Probes, Eugene, OR).

### 4.7. Detection of intracellular calcium concentration ([Ca^2+^]i)

The [Ca^2+^]i was detected with Fluo-3 AM fluorescence dye, using a modification of the previously reported method [31, 66]. Briefly, cells were co-treated with MG and glucose, harvested and washed, and then loaded with 6 μM Fluo-3 AM in standard medium (140 mM NaCl, 5 mM KCl, 1 mM MgCl_2_, 5.6 mM glucose, 1.5 mM CaCl_2_, and 20 mM Hepes, pH of 7.4). After 30 min, the cells were washed three times with PBS and then resuspended in standard medium or Ca^2+^-free standard medium. The fluorescence intensity of Fluo-3 was determined using a fluorescence spectrophotometer (Hitachi, F-2000; excitation at 490 nm, emission at 526 nm).

### 4.8. Detection of intracellular NO content

The DAF-2DA fluorescence dye was used to detect intracellular NO, according to a modification of the previously reported method [31, 67]. Briefly, treated or control cells were collected and washed, and then incubated with 3 μM DAF-2DA. After 60 min, the cells were washed three times with PBS and the fluorescence intensity was measured by a fluorescence spectrophotometer (Hitachi, F-2000; excitation at 485 nm, emission at 515 nm).

### 4.9. Caspase activity assays

Caspase-9 activity was assayed using the Colorimetric Caspase-9 Assay Kit (Calbiochem, CA). Caspase-3 activity was measured using the Z-DEVD-AFC fluorogenic substrate, as previously described [68, 69].

### 4.10. Real-time RT-PCR assay

Total RNA was extracted with the TRIzol reagent (Life Technologies) and purified with an RNeasy Mini kit (Qiagen), according to the manufacturers’ protocols. Real-time PCR was carried out with an ABI 7000 Prism Sequence Detection System (Applied Biosystems). The β-actin mRNA levels were quantified as an endogenous control, and used for normalization. The primers used for PCR were as follows: p53, 5′-CCC ATC CTC ACC ATC ATC AC-3′ and 5′-GTC AGT GGG GAA CAA GAA GTG-3′; p21, 5′-GCC GAA GTC AGT TCC TTG TGG A-3′ and 5′-GTG GGC GGA TTA GGG CTT-3′.

### 4.11. siRNA knockdown

Lipofectamine was used to transfect mononuclear cells with 150 nM of siRNA for targeting against p53 (5′-GACUCCAGUGGUAAUCUACTT-3′; sip53) or a scrambled control duplex (5′-GCGCGCUUUGUAGGAUUCG-3′; siScr). Twenty-four hours post-transfection, fresh culture medium was added, and the cells were treated with or without 5 μM MG and 15 mM glucose for another 24 h.

### 4.12. Statistics

Data were analyzed using one-way ANOVA, and differences were evaluated using a two tailed Student’s t-test and analysis of variance. P < 0.05 was considered significant.

## References

[1] Okado A, Kawasaki Y, Hasuike Y, Takahashi M, Teshima T, Fujii J, Taniguchi N (1996). Induction of apoptotic cell death by methylglyoxal and 3-deoxyglucosone in macrophagederived cell lines. Biochem. Biophys. Res. Commun.

[2] Ho FM, Liu SH, Liau CS, Huang PJ, Lin-Shiau SY (2000). High glucose-induced apoptosis in human endothelial cells is mediated by sequential activations of c-Jun NH(2)-terminal kinase and caspase-3. Circulation.

[3] Chan WH (2005). Effect of resveratrol on high glucose-induced stress in human leukemia K562 cells. J. Cell. Biochem.

[4] Hsuuw YD, Chang CK, Chan WH, Yu JS (2005). Curcumin prevents methylglyoxal-induced oxidative stress and apoptosis in mouse embryonic stem cells and blastocysts. J. Cell. Physiol.

[5] Chan WH, Wu HJ (2008). Methylglyoxal and high glucose co-treatment induces apoptosis or necrosis in human umbilical vein endothelial cells. J. Cell. Biochem.

[6] Qin S, Minami Y, Kurosaki T, Yamamura H (1997). Distinctive functions of Syk and Lyn in mediating osmotic stress- and ultraviolet C irradiation-induced apoptosis in chicken B cells. J. Biol. Chem.

[7] Chan WH, Yu JS, Yang SD (1999). PAK2 is cleaved and activated during hyperosmotic shockinduced apoptosis via a caspase-dependent mechanism: evidence for the involvement of oxidative stress. J. Cell. Physiol.

[8] Kasai H, Kumeno K, Yamaizumi Z, Nishimura S, Nagao M, Fujita Y, Sugimura T, Nukaya H, Kosuge T (1982). Mutagenicity of methylglyoxal in coffee. Gann.

[9] Baden T, Yamawaki H, Saito K, Mukohda M, Okada M, Hara Y (2008). Telmisartan inhibits methylglyoxal-mediated cell death in human vascular endothelium. Biochem. Biophys. Res. Commun.

[10] Phillips SA, Thornalley PJ (1993). The formation of methylglyoxal from triose phosphates. Investigation using a specific assay for methylglyoxal. Eur. J. Biochem.

[11] Ahmed MU Brinkmann Frye E Degenhardt TP Thorpe SR Baynes JW N-epsilon-(carboxyethyl)lysine, a product of the chemical modification of proteins by methylglyoxal, increases with age in human lens proteins Biochem J 1997 324(Pt 2)565 570 9182719 10.1042/bj3240565 PMC1218467

[12] Fosmark DS, Torjesen PA, Kilhovd BK, Berg TJ, Sandvik L, Hanssen KF, Agardh CD, Agardh E (2006). Increased serum levels of the specific advanced glycation end product methylglyoxal-derived hydroimidazolone are associated with retinopathy in patients with type 2 diabetes mellitus. Metabolism.

[13] Mostafa AA, Randell EW, Vasdev SC, Gill VD, Han Y, Gadag V, Raouf AA, El Said H (2007). Plasma protein advanced glycation end products, carboxymethyl cysteine, and carboxyethyl cysteine, are elevated and related to nephropathy in patients with diabetes. Mol. Cell. Biochem.

[14] Wu L (2006). Is methylglyoxal a causative factor for hypertension development?. Can. J. Physiol. Pharmacol.

[15] Shipanova IN, Glomb MA, Nagaraj RH (1997). Protein modification by methylglyoxal: chemical nature and synthetic mechanism of a major fluorescent adduct. Arch. Biochem. Biophys.

[16] Uchida K, Khor OT, Oya T, Osawa T, Yasuda Y, Miyata T (1997). Protein modification by a Maillard reaction intermediate methylglyoxal. Immunochemical detection of fluorescent 5-methylimidazolone derivatives in vivo. FEBS Lett.

[17] Yim HS, Kang SO, Hah YC, Chock PB, Yim MB (1995). Free radicals generated during the glycation reaction of amino acids by methylglyoxal. A model study of protein-cross-linked free radicals. J. Biol. Chem.

[18] Wu HJ, Chan WH (2007). Genistein protects methylglyoxal-induced oxidative DNA damage and cell injury in human mononuclear cells. Toxicol. In Vitro.

[19] Chan WH, Wu HJ (2006). Protective effects of curcumin on methylglyoxal-induced oxidative DNA damage and cell injury in human mononuclear cells. Acta Pharmacol. Sin.

[20] Messier C, Gagnon M (1996). Glucose regulation and cognitive functions: relation to Alzheimer’s disease and diabetes. Behav. Brain Res.

[21] Gerozissis K (2003). Brain insulin: regulation, mechanisms of action and functions. Cell. Mol. Neurobiol.

[22] Ota K, Nakamura J, Li W, Kozakae M, Watarai A, Nakamura N, Yasuda Y, Nakashima E, Naruse K, Watabe K, Kato K, Oiso Y, Hamada Y (2007). Metformin prevents methylglyoxal-induced apoptosis of mouse Schwann cells. Biochem. Biophys. Res. Commun.

[23] Okouchi M, Okayama N, Aw TY (2005). Hyperglycemia potentiates carbonyl stress-induced apoptosis in naive PC-12 cells: relationship to cellular redox and activator protease factor-1 expression. Curr. Neurovasc. Res.

[24] Jan CR, Chen CH, Wang SC, Kuo SY (2005). Effect of methylglyoxal on intracellular calcium levels and viability in renal tubular cells. Cell Signal.

[25] Halliwell B, Gutteridge JM (1990). Role of free radicals and catalytic metal ions in human disease: an overview. Methods Enzymol.

[26] Chan WH (2006). Ginkgolide B induces apoptosis and developmental injury in mouse embryonic stem cells and blastocysts. Hum. Reprod.

[27] Chan WH, Shiao NH, Lu PZ (2006). CdSe quantum dots induce apoptosis in human neuroblastoma cells via mitochondrial-dependent pathways and inhibition of survival signals. Toxicol. Lett.

[28] Ekmekcioglu S, Tang CH, Grimm EA (2005). NO news is not necessarily good news in cancer. Curr. Cancer Drug Targets.

[29] Zhou J, Brune B (2005). NO and transcriptional regulation: from signaling to death. Toxicology.

[30] Rao CV (2004). Nitric oxide signaling in colon cancer chemoprevention. Mutat. Res.

[31] Lu Z, Tao Y, Zhou Z, Zhang J, Li C, Ou L, Zhao B (2006). Mitochondrial reactive oxygen species and nitric oxide-mediated cancer cell apoptosis in 2-butylamino-2-demethoxyhypocrellin B photodynamic treatment. Free Radic. Biol. Med.

[32] Nazarewicz RR, Zenebe WJ, Parihar A, Larson SK, Alidema E, Choi J, Ghafourifar P (2007). Tamoxifen induces oxidative stress and mitochondrial apoptosis via stimulating mitochondrial nitric oxide synthase. Cancer Res.

[33] Ghafourifar P, Cadenas E (2005). Mitochondrial nitric oxide synthase. Trends Pharmacol. Sci.

[34] Brookes PS (2004). Mitochondrial nitric oxide synthase. Mitochondrion.

[35] Dennis J, Bennett JP (2003). Interactions among nitric oxide and Bcl-family proteins after MPP+ exposure of SH-SY5Y neural cells I: MPP+ increases mitochondrial NO and Bax protein. J. Neurosci. Res.

[36] Elfering SL, Sarkela TM, Giulivi C (2002). Biochemistry of mitochondrial nitric-oxide synthase. J. Biol. Chem.

[37] Dedkova EN, Ji X, Lipsius SL, Blatter LA (2004). Mitochondrial calcium uptake stimulates nitric oxide production in mitochondria of bovine vascular endothelial cells. Am. J. Physiol. Cell. Physiol.

[38] Majno G, Joris I (1995). Apoptosis, oncosis, and necrosis. An overview of cell death. Am. J. Pathol.

[39] Schwartz SM, Bennett MR (1995). Death by any other name. Am. J. Pathol.

[40] Chan WH, Yu JS, Yang SD (2000). Apoptotic signalling cascade in photosensitized human epidermal carcinoma A431 cells: involvement of singlet oxygen, c-Jun N-terminal kinase, caspase-3 and p21-activated kinase 2. Biochem. J.

[41] Chan WH, Wu HY, Chang WH (2006). Dosage effects of curcumin on cell death types in a human osteoblast cell line. Food Chem. Toxicol.

[42] Chan WH, Chang YJ (2006). Dosage effects of resveratrol on ethanol-induced cell death in the human K562 cell line. Toxicol. Lett.

[43] Thompson CB (1995). Apoptosis in the pathogenesis and treatment of disease. Science.

[44] Alison MR, Sarraf CE (1994). Liver cell death: patterns and mechanisms. Gut.

[45] Bonfoco E, Krainc D, Ankarcrona M, Nicotera P, Lipton SA (1995). Apoptosis and necrosis: two distinct events induced, respectively, by mild and intense insults with N-methyl-D-aspartate or nitric oxide/superoxide in cortical cell cultures. Proc. Natl. Acad. Sci. USA.

[46] Hampton MB, Orrenius S (1997). Dual regulation of caspase activity by hydrogen peroxide: implications for apoptosis. FEBS Lett.

[47] Eguchi Y, Shimizu S, Tsujimoto Y (1997). Intracellular ATP levels determine cell death fate by apoptosis or necrosis. Cancer Res.

[48] Leist M, Single B, Castoldi AF, Kuhnle S, Nicotera P (1997). Intracellular adenosine triphosphate (ATP) concentration: a switch in the decision between apoptosis and necrosis. J. Exp. Med.

[49] Richter C, Schweizer M, Cossarizza A, Franceschi C (1996). Control of apoptosis by the cellular ATP level. FEBS Lett.

[50] Cosentino F, Hishikawa K, Katusic ZS, Luscher TF (1997). High glucose increases nitric oxide synthase expression and superoxide anion generation in human aortic endothelial cells. Circulation.

[51] Baumgartner-Parzer SM, Wagner L, Pettermann M, Grillari J, Gessl A, Waldhausl W (1995). High-glucose–triggered apoptosis in cultured endothelial cells. Diabetes.

[52] Lee C, Yim MB, Chock PB, Yim HS, Kang SO (1998). Oxidation-reduction properties of methylglyoxal-modified protein in relation to free radical generation. J. Biol. Chem.

[53] Oya T, Hattori N, Mizuno Y, Miyata S, Maeda S, Osawa T, Uchida K (1999). Methylglyoxal modification of protein. Chemical and immunochemical characterization of methylglyoxalarginine adducts. J. Biol. Chem.

[54] Formigli L, Papucci L, Tani A, Schiavone N, Tempestini A, Orlandini GE, Capaccioli S, Orlandini SZ (2000). Aponecrosis: morphological and biochemical exploration of a syncretic process of cell death sharing apoptosis and necrosis. J. Cell. Physiol.

[55] Kass GE Eriksson JE Weis M Orrenius S Chow SC Chromatin condensation during apoptosis requires ATP Biochem J 1996 318(Pt 3)749 752 8836114 10.1042/bj3180749 PMC1217681

[56] Almeida RD, Manadas BJ, Carvalho AP, Duarte CB (2004). Intracellular signaling mechanisms in photodynamic therapy. Biochim. Biophys. Acta.

[57] Inanami O, Yoshito A, Takahashi K, Hiraoka W, Kuwabara M (1999). Effects of BAPTA-AM and forskolin on apoptosis and cytochrome c release in photosensitized Chinese hamster V79 cells. Photochem. Photobiol.

[58] Monteiro HP, Silva EF, Stern A (2004). Nitric oxide: a potential inducer of adhesion-related apoptosis–anoikis. Nitric Oxide.

[59] Li CQ, Wogan GN (2005). Nitric oxide as a modulator of apoptosis. Cancer Lett.

[60] Gomes ER, Almeida RD, Carvalho AP, Duarte CB (2002). Nitric oxide modulates tumor cell death induced by photodynamic therapy through a cGMP-dependent mechanism. Photochem. Photobiol.

[61] Li CQ, Robles AI, Hanigan CL, Hofseth LJ, Trudel LJ, Harris CC, Wogan GN (2004). Apoptotic signaling pathways induced by nitric oxide in human lymphoblastoid cells expressing wild-type or mutant p53. Cancer Res.

[62] Okada H, Mak TW (2004). Pathways of apoptotic and non-apoptotic death in tumour cells. Nat. Rev. Cancer.

[63] Menon RP, Pillai S, Sudhakaran PR (1993). Binding of collagen causes intracellular mobilization of calcium in human mononuclear cells. Biochem. Mol. Biol. Int.

[64] Wu HJ, Chan WH (2006). Genistein protects methylglyoxal-induced oxidative DNA damage and cell injury in human mononuclear cells. Toxicol In Vitro.

[65] Behl C, Davis JB, Lesley R, Schubert D (1994). Hydrogen peroxide mediates amyloid beta protein toxicity. Cell.

[66] Aoshima H, Satoh T, Sakai N, Yamada M, Enokido Y, Ikeuchi T, Hatanaka H (1997). Generation of free radicals during lipid hydroperoxide-triggered apoptosis in PC12h cells. Biochim. Biophys. Acta.

[67] Nakatsubo N, Kojima H, Kikuchi K, Nagoshi H, Hirata Y, Maeda D, Imai Y, Irimura T, Nagano T (1998). Direct evidence of nitric oxide production from bovine aortic endothelial cells using new fluorescence indicators: diaminofluoresceins. FEBS Lett.

[68] Chan WH, Wu CC, Yu JS (2003). Curcumin inhibits UV irradiation-induced oxidative stress and apoptotic biochemical changes in human epidermoid carcinoma A431 cells. J. Cell. Biochem.

[69] Hsieh YJ, Wu CC, Chang CJ, Yu JS (2003). Subcellular localization of Photofrin determines the death phenotype of human epidermoid carcinoma A431 cells triggered by photodynamic therapy: when plasma membranes are the main targets. J. Cell. Physiol.

